# Anomalous Systemic Arterial Supply to the Basal Segment of the Lung (ABLL) in the Right Lower Lobe: Preoperative Embolization and Thoracoscopic Surgery

**DOI:** 10.7759/cureus.82152

**Published:** 2025-04-12

**Authors:** Takayuki Hatakeyama, Takahiro Homma, Kanji Otsubo, Hiroki Sakai, Hiroyuki Kimura, Tomoyuki Miyazawa, Hideki Marushima, Koji Kojima, Hisashi Saji, Natsumi Toyoda, Yoshiya Sugiura, Nobuyuki Ohike

**Affiliations:** 1 Thoracic Surgery, St. Marianna University School of Medicine, Kawasaki, JPN; 2 Pathology, St. Marianna University School of Medicine, Kawasaki, JPN

**Keywords:** abll, anomalous systemic arterial supply to the basal segment of the lung, bleeding, embolization, vascular plug, video-assisted thoracoscopic surgery

## Abstract

Anomalous systemic arterial supply to the basal segment of the lung (ABLL) is a rare congenital anomaly. Surgical resection is the standard treatment to prevent complications like hemoptysis. Right-sided ABLL, often characterized by longer and more complex vascular anatomy, poses a higher risk of bleeding. Preoperative planning and embolization techniques are crucial for successful surgical outcomes. This case of right-sided ABLL is presented to emphasize the importance of these strategies.

A 55-year-old woman experienced right lower abdominal pain. Incidental findings on computed tomography revealed an abnormal vessel supplying segment 10 of the right lower lobe, diagnosed as ABLL. To minimize intraoperative hemorrhage, preoperative embolization with a vascular plug was performed. Video-assisted thoracoscopic surgery was subsequently undertaken. The aberrant artery was divided with an endoscopic stapler, and a wedge resection of the affected lung was performed.

This case report demonstrates the benefits of combining embolization and surgical resection for right-sided ABLL. This approach would reduce the risk of intraoperative bleeding.

## Introduction

Anomalous systemic arterial supply to the normal basal segment of the lung (ABLL) is a rare congenital anomaly, with the left lower lobe being a common site of abnormality. Surgical resection of the aberrant artery and affected lung is the gold standard treatment for ABLL [1], which aims to minimize potential complications such as hemoptysis. The abnormal vessels on the right side are generally longer than those on the left side. This increases the risk of intraoperative bleeding or postoperative aneurysm formation in ABLLs. Consequently, ABLL surgery can be complex [2-5].

Here, we report a surgical case involving a 55-year-old female with a right-sided ABLL. This case highlights the importance of preoperative planning and the use of embolization techniques in ABLL surgery.

## Case presentation

**Figure 1:**
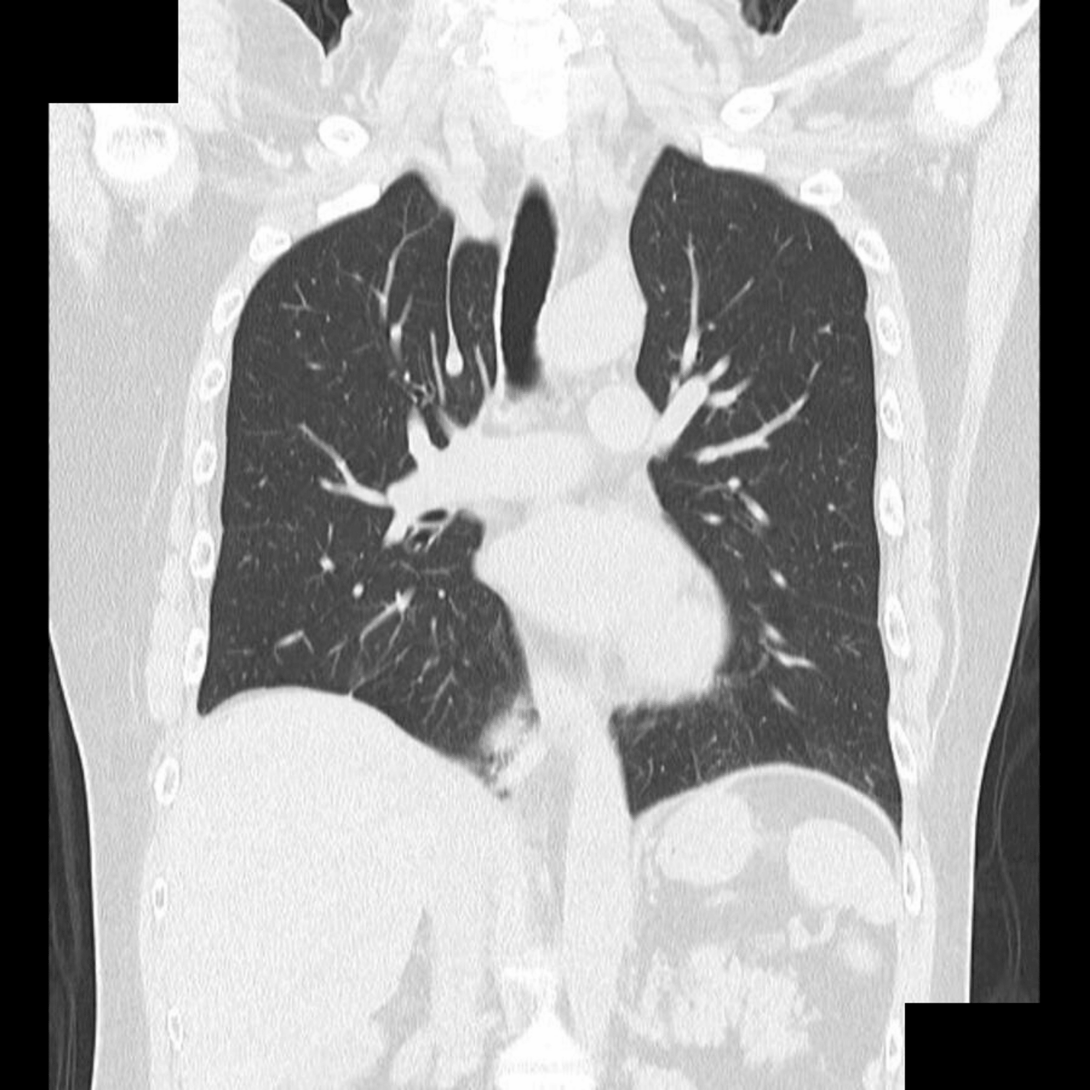
CT showed an abnormal mass in the right lower lobe, which connects to the descending aorta.

**Figure 2:**
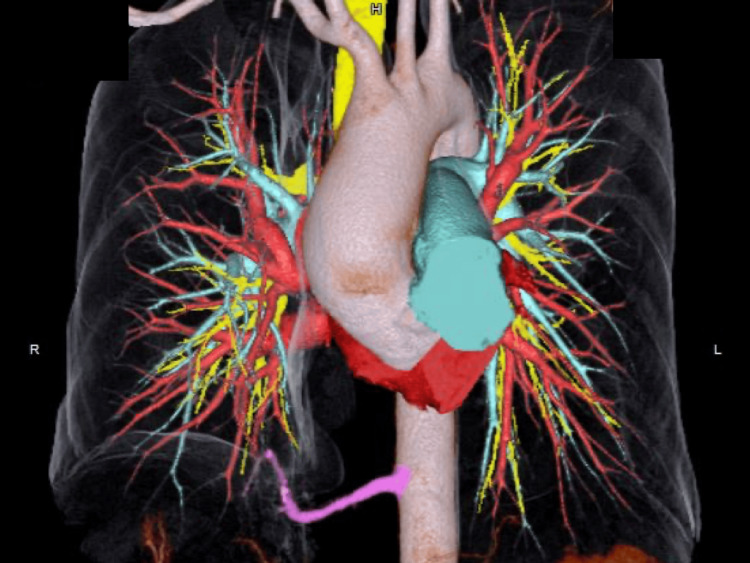
Enhanced CT revealed an abnormal artery supplying segment 10 of the right lower lobe branching from the descending aorta. The course of the bronchi was normal.

A 55-year-old female presented to the hospital with complaints of right lower abdominal pain. The patient had no past medical history except for urethral stones. Computed tomography (CT) revealed a urethral stone and incidentally revealed an abnormal shadow in the right lower chest (Figure 1). Enhanced CT revealed abnormal vessels supplying segment 10 of the right lower lobe, branching from the descending aorta, with no abnormalities in the course of the bronchi (Figure 2). The diagnosis was ABLL. Blood tests and electrocardiograms revealed no abnormalities, and spirometry indicated a mild restrictive ventilatory disorder (vital capacity percentage (%VC) 65.31%, forced expiratory volume in 1 second percentage (FEV1%) 75.89%).

**Figure 3:**
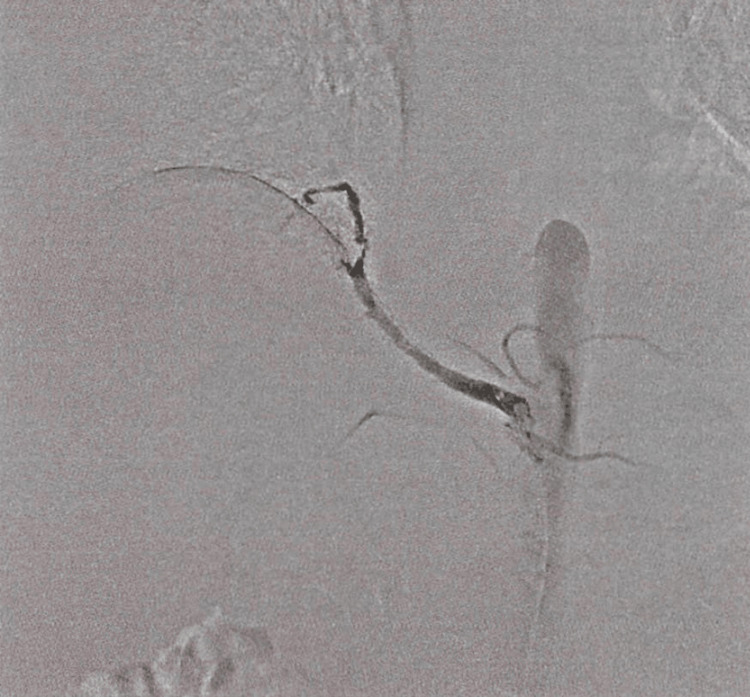
Angiography image showing an abnormal artery branching from the descending aorta, which supplies segment 10 of the right lower lobe.

**Figure 4:**
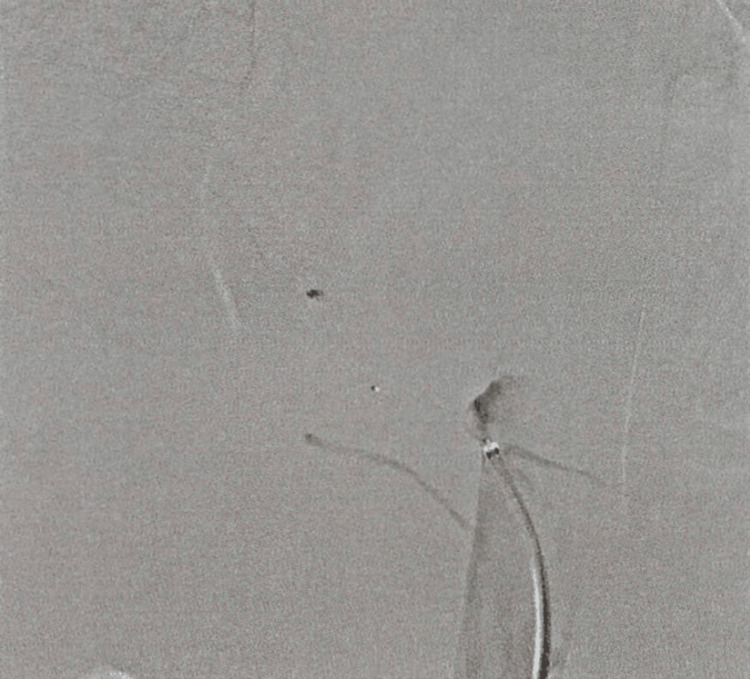
Complete occlusion after the placement of the vascular plug.

Video-assisted thoracoscopic surgical resection for ABLLs was planned. To prevent intraoperative hemorrhage, a vascular plug was first employed for embolization the day before surgery (Figures 3-4). During the operation, three ports were placed. One in the sixth intercostal space and two in the eighth intercostal space. The aberrant artery was divided with the pulmonary ligament using an endoscopic stapler near the branching point, leaving the vascular plug. Finally, a wedge resection of the reddish part of the right lower lobe was performed. The patient was discharged five days after the surgery. At the outpatient follow-up, the patient progressed without any complications.

**Figure 5:**
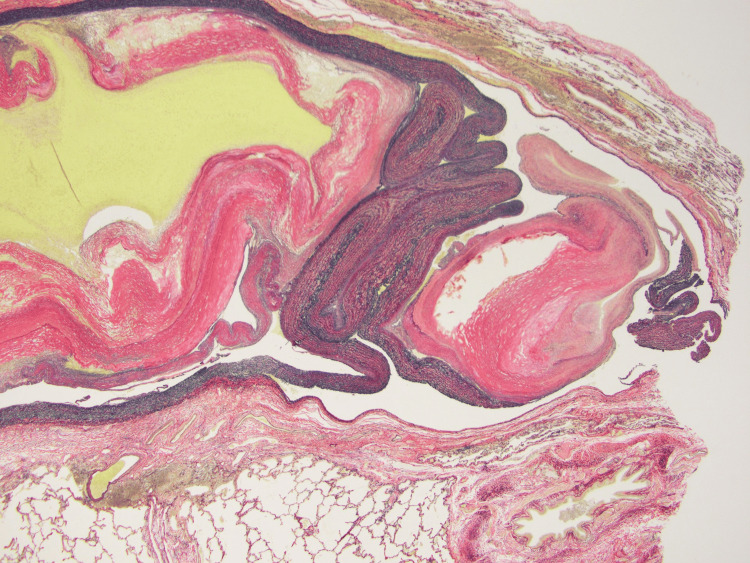
Pathology revealed cross-sections of abnormal arteries (EVG stain (×10)). Unlike pulmonary arteries, they are not accompanied by the bronchus. EVG stain: Elastica van Gieson stain

**Figure 6:**
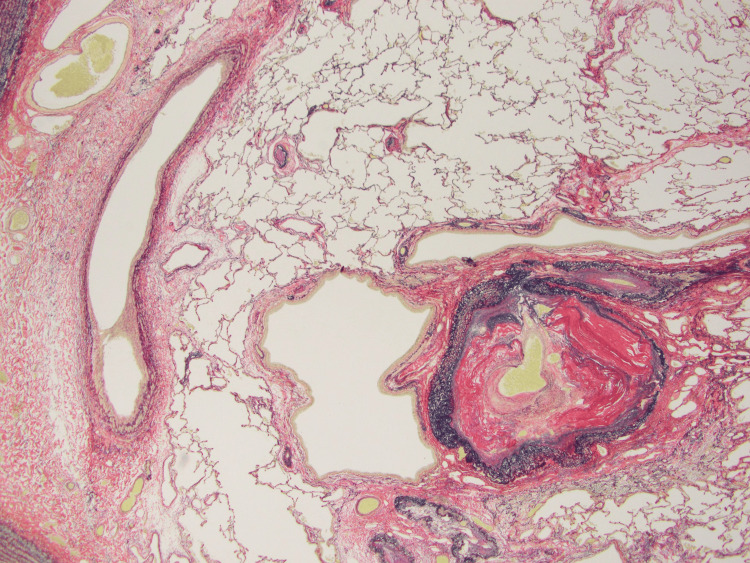
The lumens of the normal pulmonary arteries were narrowed due to high blood pressure (EVG stain (×10)). EVG stain: Elastica van Gieson stain

Pathology revealed abnormal arteries inside the right lower lobe (Figure 5). Unlike pulmonary arteries, they are not aligned with the bronchus. Additionally, the lumens of normal pulmonary arteries are narrowed because of high blood pressure (Figure 6).

## Discussion

ABLL comprises 0.15% to 6% of all congenital pulmonary malformations [6]. The most likely theory of ABLL is a persistent embryonic connection between the aorta and pulmonary parenchyma due to failure to regress from the primitive aortic branches to the developing lung bud [7]. ABLL is thus considered part of the spectrum of pulmonary sequestration, specifically classified as Pryce type I. Most cases of ABLL are localized in the basal segments of the left lower lobe (91.4%) [8].

While most ABLL patients remain asymptomatic, the condition can present later in life, often with hemoptysis as the primary symptom. The potential for life-threatening bleeding highlights the importance of timely and appropriate intervention. Previous reports have shown either surgical resection or embolization. Surgical resection of the aberrant artery and resection of the affected lung are recognized as the gold standards for treating ABLLs. Embolization is considered for inoperable cases [9]. However, coil embolization is not recommended in cases where the inner vessel diameter is 10 mm or greater because of the risk of incomplete occlusion and coil migration. The median diameter of the aberrant artery has been reported to be 10.0 mm (range: 4.0-20.0 mm) [9].

This patient underwent combined embolization with surgery. Embolization using a vascular plug was performed first, followed by thoracoscopic surgery. The diameter of the abnormal vessel was approximately 5 mm, so a Vascular Plug 4 (8 × 13.5 mm) was chosen. The vascular plug diameter was 1.5 times greater than the diameter of the abnormal vessel. Preoperative embolization is considered a crucial and preventive step, as surgical treatment carries significant risks. These include inadvertent intraoperative injury to abnormal vessels and the potential formation of aneurysms in the future. This preventive approach is particularly important when the target site is located deep behind other organs, making ligation technically challenging.

This case emphasizes the importance of combining embolization with surgical resection, as this approach enhances procedural safety and promotes faster recovery, regardless of the surgeon’s experience or the specific surgical site. As more cases are documented and techniques continue to evolve, patient outcomes for this rare anomaly are expected to improve. Further research is needed to establish standardized protocols that can help clinicians manage similar cases more effectively.

## Conclusions

This case demonstrates the benefits of combining embolization and surgical resection for right-side ABLL. This approach would reduce the risk of intraoperative bleeding, which ensures a safe procedure and fast recovery.
